# A review and new perspective on oral bacteriophages: manifestations in the ecology of oral diseases

**DOI:** 10.1080/20002297.2024.2344272

**Published:** 2024-05-01

**Authors:** Xinyu Guo, Xiaowan Wang, Jia Shi, Jiayi Ren, Jumei Zeng, Jinquan Li, Yuqing Li

**Affiliations:** a State Key Laboratory of Oral Diseases, National Clinical Research Center for Oral Diseases, West China Hospital of Stomatology, Sichuan University, Chengdu, China; b West China School of Public Health and West China Fourth Hospital, Sichuan University, Chengdu, China; c State Key Laboratory of Agricultural Microbiology, College of Biomedicine and Health, College of Food Science and Technology, Huazhong Agricultural University, Wuhan, China; d Center for Archaeological Science, Sichuan University, Chengdu, China

**Keywords:** Bacteriophage, oral disease, *Streptococcus mutans*, *Fusobacterium nucleatum*, *Enterococcus faecalis*

## Abstract

**Objective:**

To explore the manifestations of bacteriophages in different oral disease ecologies, including periodontal diseases, dental caries, endodontic infections, and oral cancer, as well as to propel phage therapy for safer and more effective clinical application in the field of dentistry.

**Methods:**

In this literature review, we outlined interactions between bacteriophages, bacteria and even oral cells in the oral ecosystem, especially in disease states. We also analyzed the current status and future prospects of phage therapy in the perspective of different oral diseases.

**Results:**

Various oral bacteriophages targeting at periodontal pathogens as *Porphyromonas gingivalis*, *Fusobacterium nucleatum*, *Treponema denticola* and *Aggregatibacter actinomycetemcomitans*, cariogenic pathogen *Streptococcus mutans*, endodontic pathogen *Enterococcus faecalis* were predicted or isolated, providing promising options for phage therapy. In the realm of oral cancer, aside from displaying tumor antigens or participating in tumor-targeted therapies, phage-like particle vaccines demonstrated the potential to prevent oral infections caused by human papillomaviruses (HPVs) associated with head-and-neck cancers.

**Conclusion:**

Due to their intricate interactions with bacteria and oral cells, bacteriophages are closely linked to the progression and regression of diverse oral diseases. And there is an urgent need for research to explore additional possibilities of bacteriophages in the management of oral diseases.

## Introduction

Oral microbiome plays a vital role in maintaining oral health. Several reviews have addressed the relationship between microbial dysbiosis and oral diseases. Oral microorganisms are involved in the development and progression of various oral diseases and even certain systemic diseases [1–4]. Moreover, oral microbiome is strongly related to tumor progression *in situ* or metastasis, especially oral anaerobes such as *Fusobacterium nucleatum* and *Porphyromonas gingivalis* [5]. It was recently discovered that viruses have been grossly underestimated in oral dysbiosis [6]. According to Ly et al., viruses in oral biofilms dramatically changed in patients with periodontal disease compared to healthy individuals, suggesting that viruses could be an indicator for determining oral health status [7]. Both eukaryotic viruses and bacteriophages (prokaryotic viruses) can be detected in the oral virome of healthy individuals; however, since bacteriophages are considerably more prevalent in the oral cavity [8,9], this review will concentrate only on them. Other reviews delved into the hazards of bacteriophages in the progression of periodontitis and the benefits of using them for therapeutic purposes [10,11]. Some recent studies also investigated the balance between bacteriophages and oral microbiome. Based on bacterial taxonomy, Szafrański et al. comprehensively summarized the effects of oral bacteriophages and their lysins on complex biofilms [12].

With the growing clinical challenge of antibiotic resistance, phage therapy for oral infectious diseases has also attracted renewed attention. It has been emphasized that phages directed against *S. mutans* may be used as a possible caries prevention strategy [13]. Furthermore, research on phage therapy for endodontic and periapical infections is in full swing [14–16]. Steier et al. reviewed oral bacteriophages concerning their structure, mode of action on bacteria in biofilms, and possible future applications in dentistry [17].

This work proposes a review and new perspective on recognizing oral bacteriophages, i.e. their manifestations in different oral disease ecologies. We address the characterization of these bacteriophages and their dynamics with ecological sites in each oral disease to assist clinicians in gaining a more comprehensive understanding of oral bacteriophages and implementing safe and effective phage therapies in dentistry at an early stage.

On the source of this narrative review, we primarily performed literature search in the PubMed database with the terms of ‘oral bacteriophages’, ‘oral diseases’ and ‘phage therapy’ published mainly in the last five years. Furthermore, the citations of all retrieved articles were checked for additional pertinent references.

## Main text

The viruses in the oral cavity have caused increasing concern. As we have recognized that viral communities were prevalent in the saliva and subgingival or supragingival biofilms in healthy individuals and patients, composed predominantly of bacteriophages [8,18]. Moreover, previous studies based on saliva and dental plaque demonstrated that viruses were stable and individualized members of the oral ecosystem [5,19]. Despite the relative rarity, Zhang et al. suggested that there existed a general core of oral viruses, primarily *Streptococcus* phages, and *Herpesviruses* of the eukaryotic virus family [20].

## Complex interactions between bacteria, bacteriophages, and oral cells

### Bacteriophages affect bacteria in diverse replication cycles

**Figure 1.**
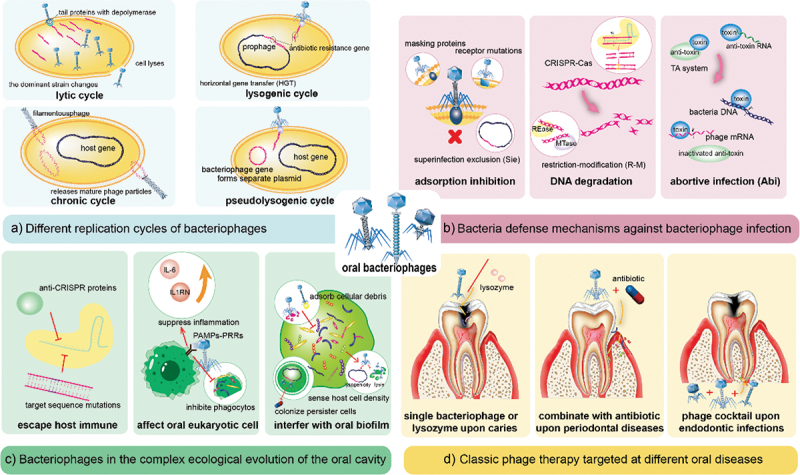
Interactions between bacteriophages, bacteria, oral cells, and classical phage therapy in dentistry.

Previous reviews have summarized that bacteriophages could influence bacteria through various processes because they can replicate through four different mechanisms: the lytic, lysogenic, chronic, or pseudolysogenic cycles [21] (Figure 1). At the infection and invasion stage, bacteriophages take advantage of the tail proteins with depolymerase or produce various enzymes to disrupt biofilms [22]. After entering the host cytoplasm, virulent bacteriophages replicate through the lytic cycle and lyse the host bacteria [23], hastening the transformation of the dominant strain. Simultaneously, temperate bacteriophages form prophages to integrate into host genome. Scholars have concluded that this extensive HGT offers the potential for the dissemination of pathogenic islands (PAI), particularly those linked to antibiotic resistance [24,25], which is consistent with the discovery that people in close contact with each other shared a limited population of oral viruses and exhibit more analogous antibiotic resistance [6,18].

(a) Different replication cycles of bacteriophages: Lysis cycles can cause host cells to lyse and alter biofilm dominant colonization, while lysogenic and pseudolysogenic cycles can occur horizontal gene transfer, including virulence factors and antibiotic resistance genes.

(b) Bacterial defense mechanisms against bacteriophage infection: Bacteria inhibit phage adsorption by masking receptors, receptor mutations, and the superinfection exclusion (Sie) system. Bacteria degrade exogenous phage DNA using clustered regularly interspaced short palindromic repeats (CRISPR)-Cas and restriction-modification (R-M) systems. They mainly use the toxin-antitoxin system to deliver abortive infection (Abi) system functions.

(c) Bacteriophages in the complex ecological evolution of the oral cavity: Bacteriophages can escape the host immune system through anti-CRISPR proteins or target sequence mutations. Bacteriophages would affect oral eukaryotic cells by activating pathogen-associated molecular patterns (PAMPs)-pattern-recognition receptors (PRRs) pathway, inducing anti-inflammatory mediators like IL-6 and IL1RN, or inhibiting phagocytosis. In oral biofilms, bacteriophages have unique evolutionary properties, including adsorbing extracellular polysaccharides or cellular debris to deposit bacterial high-density regions, corresponding quorum sensing to alter lysis-lysogenicity decisions, and permanently colonizing persister cells to avoid antibiotic attacks.

(d) Classic phage therapy targets different oral bacteria: monophages or lysozymes upon caries, combining with antibiotics upon periodontal diseases, and phage cocktail upon endodontic infections.

### Bacteria and bacteriophages develop varied mutual defense mechanisms

Over the long period of co-evolution, bacteria have developed various defense mechanisms against phage infection. These mechanisms include adsorption inhibition, degrading phage DNA [26], superinfection exclusion (Sie), restriction-modification (R-M) systems, clustered regularly interspaced short palindromic repeats (CRISPR)-Cas, and abortive infection (Abi) systems [27–29] (Figure 1).

Concurrently, bacteriophages have evolved multiple strategies to circumvent adaptive immunity as Malone et al. review concluded, including anti-CRISPR proteins, nuclear-like protective structures, and target sequence mutations (Figure 1) [30]. In oral cells, bacteriophages mainly induce anti-inflammatory mediators such as IL-6 and IL1RN to regulate innate and adaptive immunity, thus indirectly affecting cellular signaling and physiological processes [31]. According to Zou et al., bacteriophages can regulate gene expression, adsorb lipopolysaccharide (LPS), and prevent macrophages from phagocytosing bacteria by activating the pathogen-associated molecular patterns (PAMPs)-pattern recognition receptors (PRRs) pathway [32]. These discoveries help clarify the intricate interactions between bacteriophages, bacteria, and the oral cavity, as well as the ecological dynamics underlying the rapid evolution of phages.

### Bacteriophages within oral biofilms exhibit more complicated features

The interactions between bacteriophages and bacteria at the specified *loci* have sparked considerable interest with the advent of ecohistology. Hansen et al. strongly stressed the importance of biofilms in bacteriophages studies, as they are associated with antibiotic resistance and responsible for most chronic infections [33]. Using *in vitro* cultures, Correa et al. recently proposed that viral infection in biofilms had a continuum that varied from effective infection and ineffective lysis caused by virulent phages to chronic infection and persistent lysogenic infection via temperate phages [34].

Oral biofilms called dental plaques are closely related to infectious diseases of the oral cavity, such as periodontitis, caries, and apical periodontitis [30,31], and even potentially impact systemic health [35]. Using simulation models, Bull et al. revealed that bacteriophages and bacteria possess more sophisticated dynamics in spatially structured biofilms. Certain bacteriophages inadvertently attach themselves to extracellular polysaccharides or cellular debris, serving as refuges for both phages and bacteria, which ultimately raises the equilibrium cell density within the system [36]. The host bacterial population likewise restricts bacteriophages growth, as phages could adjust their lysis-lysogenicity decisions based on host density in response to quorum sensing [37]. Furthermore, bacteriophages could also permanently colonize persister cells to avoid antibiotic attacks, hence Abedon et al. argued that it may be a mechanism for treatment failure in patients with chronic or recurrent infections [38]. More interestingly, the oral phage community is a potential reservoir of bacterial PAI. For instance, *Streptococcus mitis* phage SM1, localized in the oropharynx, has the platelet binding factors pblA and pblB genes [39]. Although bacteriophage studies on oral biofilms are still in fancy, continued work should consider the overall ecological evolution of the oral cavity.

## Profiles of phage therapy in dentistry

Phage therapy is a popular strategy for dealing with bacteria resistant to common antibiotics and antimicrobials in oral biofilms [40]. In addition to single bacteriophage, phage-derived enzymes, phage-antibiotic combinations, synthetic phages, and cocktail formulations that synergistically target multiple pathogenic bacteria are being recommended for clinical practice [41,42]. Shlezinger et al. provided a chart summarizing the benefits and risks of phage therapy and further discussed the existing applications and prospects of phage therapy in dentistry [40]. It is not the only case; Szafrański et al. reviewed the use of bacteriophages and their enzymes to control oral biofilms [12]. From the perspective of phage-mammalian immune system interactions, Zou et al. categorized the factors affecting phage therapy, including the mode of administration and dosage, the physiological state of the patient, and the biological properties of bacteriophages [32], providing a guide for phage therapy against various human diseases. The next concern is whether bacteriophages mediate adverse effects during therapy. Encouragingly, Gangwar et al. demonstrated that short-term and long-term oral administration of high doses of bacteriophage did not adversely affect rats [43]. The different disease categories below cover specific phage therapy uses (Figure 1).

While writing this review, most clinical trials or case reports on phage therapy currently registered in the Cochrane Central Register of Controlled Trials (CENTRAL) are for pulmonary infection [44], cystic fibrosis [45], diarrheal [46,47], chronic wound infections [48,49], joint infections [50,51], metabolic syndrome [52]. Although an increasing number of diseases are also being included such as chronic rhinosinusitis and Alzheimer’s Disease [53], unfortunately none of the oral diseases is among them. One aspect is that clinical trials of phages inherently face multiple challenges. Hesse et al. summarized the hurdles for commercially available phage therapeutics as relevant pharmacological and toxicological studies had yet to propose a universal model; the financial burden of large-scale clinical trials was costly; and there was difficulty in predicting the phage host range and the specific immune response it induced *in vivo* [54]. Hatfull et al. argued that the difficulties of phage therapy also lay in the preparation and storage of phages; the difference between the phage infection profiles and clinical strains, resistance to bacteriophages [55]. Another aspect is that some unique concerns remain about the application of phages in oral diseases: whether orally administered phages can tolerate the temperature fluctuating from 0°C to 70°C [56] and maintain stability, and can effetive phages approach deep-seated pathogens in complicated oral biofilms such as subgingival plaque? Hopefully, viral metagenomics can aid in determining more genetic information about unisolated phages, even predicting their target hosts, exploring how phages are involved in oral diseases procession, and expanding the potential of phage engineering [31,57]. Baker et al. suggested that current *in vitro* models used for oral research could hardly recapture the interaction between oral microbiome and the host [4], so there is still far for phage therapy to become a clinical choice in dentistry.

## May phage therapy be a new attempt to manage periodontal diseases?

Periodontal diseases are broadly defined as inflammatory conditions affecting the periodontal tissues (gingiva, alveolar bone, and periodontal membrane). Alarmingly, most gingivitis patients do not receive timely diagnosis and treatment, allowing the inflammation progress into chronic periodontitis, ultimately leading to tooth loss and systemic inflammation [1]. Additionally, the evidence so far has indicated a favorable correlation between periodontal diseases and the risk of oral cancers and even systemic cancers [58,59]. As a chronic infectious disease, periodontal diseases are linked to microbial plaque dysbiosis and host immune defense imbalance [60]. Nevertheless, bacteria alone seem to fail to explain the full reasons for the devastating progression of periodontal diseases, and that is when viruses attracted researchers’ attention. Early attention has been paid to the contribution of eukaryotic viruses to periodontal diseases, such as herpesviruses, Epstein-Barr virus, and cytomegalovirus [61–63]. Bacteriophages are credited with regulating oral biofilms. Yost et al. found that sites of periodontitis progression exhibited elevated herpesvirus and bacteriophage activity compared to baseline samples [64]. Likewise, Ly et al. demonstrated a significantly higher proportion of *mycoviruses* in subgingival biofilms of patients with periodontal diseases compared to healthy individuals, suggesting that periodontal diseases favor the growth of lysogenic bacteriophages [7].

### Classical periodontal pathogens with targeted bacteriophages

*P. gingivalis* is an important member of the red microbial complex and a driver of inflammation [65], but its bacteriophage studies have not yielded anything for decades. Recently, Matrishin et al. reported that *P. gingivalis* owned phylogenetically distinct prophages that shaped host physiology, including *Alisviridae*, *Ludisviridae*, and *Nixviridae* [66]. Nevertheless, no relevant lytic bacteriophage has been isolated.

Other members of the red complex include *Tannerella forsythia* and *Treponema denticola* [67]. Szafranski et al. reported four proposed bacteriophages with oral origin associated with *T. forsythia* and 19 with *Prevotella intermedia*/*Prevotella nigrescens* by examining the IMG/VR database [31]. In addition, Mitchell et al. successfully detected lytic bacteriophage DNA from *T. denticola* and named it φtd1 [68].

As an ‘adherent’ bacterium, *F. nucleatum* forms a bridge between commensal early inhabitants and pathogenic late settlers in the subgingival plaque, contributing to the development of chronic periodontitis [69]. Isolated from saliva samples, FnpΦ02 is the first bacteriophages described to infect *F. nucleatum* [70]. Recently, Kabwe et al. discovered FNU1, a novel lytic bacteriophage against *F. nucleatum*, with the ability to intracellularly kill bacteria, significantly reducing the biofilm mass [71].

*Aggregatibacter actinomycetemcomitans* is known for its strong association with localized aggressive periodontitis [72,73]. Szafranski et al. summarized the bacteriophages targeting *A. actinomycetemcomitans* [12]. Although most of the phages are temperate, lytic AabΦ01 and AabΦ01–1 were reported by Castillo-Ruiz et al. [74]. However, their high selectivity for serotype b limited their broad clinical application.

Gram-positive anaerobic bacterium *Actinomyces naeslundii* is abundant in dental plaque and is believed to have a role in gingivitis and root surface caries. Among the numerous virulent bacteriophages isolated [12], the lytic function gene of Av-1 was successfully isolated and expressed, which consists of two holin-like genes, *holA* and *holB*, and an endolysin gene *lysA* [75]. Further studies of the specific lytic site of this endolysin will reveal why *A. naeslundii* is resistant to conventional lysozyme-like enzymes and discover effective ways to combat this bacterium.

### Bacteriophages may participate in the progression of periodontal diseases

Although the pace of research concerning lytic bacteriophage isolation from periodontal pathogens is slow, the new insight is that lysogenic bacteriophages may influence bacterial physiology and consequently participate in the progression of periodontal diseases. Using a viral macro-genomics approach, Zhang et al. recently isolated a new bacteriophage named *Siphoviridae*_29632 from patients with periodontitis. It had a significantly higher detection rate in patients with chronic periodontitis than in healthy individuals, suggesting that *Siphoviridae*_29632 was correlated with the occurrence of chronic periodontitis [76].

Bacteriophages could regulate the red complex’s growth, adhesion, and mutual competition. Yost et al. proved that *P. gingivalis* highly upregulated CRISPR-associated genes to defend against viral infection during the progression of periodontal diseases [64], which may assist its dominance in periodontal pocket biofilms. More interestingly, *T. forsythia* can deliver spacer and related Cas proteins from its CRISPR/Cas system into *P. gingivalis*, attacking the methyltransferase gene and limiting *P. gingivalis* growth [77]. The recently discovered mutant strain of *T. denticola* that lacks phage-derived genes exhibited improved growth ability and decreased motility and adhesion to gingival epithelial cells compared to the wild strain [78]. Further exploration of the connection between the conserved regions of bacteriophage genomes and bacterial pathogenicity islands may enhance the comprehension of periodontal pathogens’ physiological and pathogenic characteristics.

*A. actinomycetemcomitans* phages also bidirectionally regulate pathogenic processes. On the one hand, prophages affect bacterial virulence and aid in evading the immune response. *A. actinomycetemcomitans* serotype e strains from highly inflamed tissues, such as chronic gingivitis, could frequently detect transposable prophages likely to contain virulence factors [79]. On the other hand, bacteriophages could modulate host populations according to the internal environment. The newly isolated bacteriophage S1249 had both lysogenic and pseudolysogenic cycles, which would perform the lysogenic-lytic transformation in the presence of human serum, hence decreasing the survival of infected bacteria *in vivo* [80]. Through macro-genomics analyses, Wang et al. found that phage and bacterial composition were highly diverse in periodontally healthy patients, whereas diseased gingival samples tended to be homogeneous. Remarkably, they found the presence of cross-infecting phages (CIP), whose quantities showed a negative correlation with the main periodontal pathogens and a positive correlation with commensal bacteria [81]. These findings imply that an in-depth analysis of CIP might contribute to our current understanding of the impact of bacteriophages on bacterial community diversity and individual variability.

### Bacteriophages as a complement to conventional periodontal treatment

Given the prevalence of antimicrobial-resistant (AMR) bacteria in the oral cavity, the current view advocates distinct forms of phage therapy as a complement to traditional periodontal treatments to minimize the systemic adverse effects [82,83].

Since periodontal disease is a chronic infectious disease mediated by multiple bacteria, Pinto et al. suggested phage cocktail as an alternative to current treatments and proposed phage-based product development steps [10]. Amankwah et al. summarized the application of bacteriophages, lysozyme, and antimicrobial agents individually or in combination with bacterial biofilm formation, particularly considering that CRISPR-associated DNA nucleases encoded by temperate phages could reverse antibiotic resistance [84]. From an evidence-based standpoint, Abdulkareem et al. emphasized that, unlike broad-spectrum antibiotics, bacteriophages could eradicate dental biofilms without affecting the local ecology [83]. If the phage receptor is a bacterial virulence factor, it targets only ‘virulent’ subpopulations, and if the receptor mediates bacterial interactions, the bacteriophage disrupts intercellular interactions such as adhesion [12]. The specificity advantages of bacteriophages can be used in microbial engineering for precision medicine or to overcome antibiotic resistance. However, another challenge is that the protein capsid of the bacteriophages is susceptible to infection from external factors during transportation, leading to decreased specific infectivity. Fortunately, various encapsulated carriers (e.g. fibers, hydrogels, and particles) have been applied to phage delivery systems targeting the intestinal flora, demonstrating superiority in maintaining phage stability, protecting phage viability, and efficiently releasing the phage into the colon [85]. Although the oral cavity has different ecological characteristics compared to the colon, phage delivery systems may also function in concert with other dental materials to treat or prevent infectious disorders like periodontal diseases.

## Abundant lytic phages provide versatile options for caries treatment

As early colonizers of biofilms and members of mature biofilms, the *Streptococci* are essential to the oral ecosystem [86]. They are regarded as symbiotic and produce lactic acid, among which *S. mutans* is a key microorganism in dental caries [31]. Baker et al. showed that *S. mutans* and human herpesvirus type 4 (Epstein-Barr virus) are more common in children with dental caries [87].

### Diverse lytic bacteriophages against cariogenic bacteria

Intriguingly, bacteriophages targeting cariogenic microorganisms are still being isolated one after another. M102 was specific for serotype c strains of *S. mutans*, with a unique lysis cassette [88], while ΦAPCM01 and newly isolated SMHBZ8 inhibited *S. mutans* biofilms [89,90]. Acting as a co-aggregator in bacterial colonization, *Actinomyces spp*. were isolated from deep carious dentin [91]. Additionally, by spontaneously generating and releasing extracellular DNA from the host cell, a newly isolated linear plasmid-like phage xhp1 from *A. odontolyticus* subspecies XH001 could promote biofilm formation [92]. Bacteriophages can both lyse oral biofilms or promote biofilm formation, so their dual-sided effects may be tricky in clinical applications.

### Multiple strategies of phage therapy to combat caries

It is encouraging that investigations *in vitro* and *in vivo* have shown the effectiveness of the mentioned bacteriophages and their derivatives in preventing and treating dental caries. According to Wolfoviz et al., SMHBZ8 showed similar efficacy to conventional therapeutic dentistry in reducing *S*. *mutans* load and preventing caries development, particularly suspensions and extended-release formulations showed superior and persistent antimicrobial effects [13].

Antimicrobial peptides produced by bacteriophages are superior candidates for caries management. As an illustration, the genetically engineered peptide C16G2 exhibited the highest bactericidal capacity against *S. mutans* and *S. salivarius*, permitting their natural rivals to take the lead and establish a healthy dental community [93]. ClyR lysin, a well-known chimeric lysozyme, could selectively act on cariogenic *S. mutans* and *S. sobrinus* without affecting other harmless commensal oral bacteria. Alternatively, continuous use of ClyR could considerably decrease the dental caries rate in rat models with either single or mixed infections [94], suggesting that ClyR may be a promising agent or additive to treat dental caries. A 64-mg/L concentration of antimicrobial peptide GH12 demonstrated the most effective inhibition of oral biofilm integrity *in vivo* and significantly reduced caries scores for both sulcal and smooth surface caries at all sites [95]. More research is necessary to fully understand their anticaries effects and functions in microecological regulation to ensure a safe and efficient application of these compounds in human healthcare.

Glucosyltransferase (Gtfs) has been extensively studied as the main virulence factor of *S. mutans*. As a result, modifying the genome *loci* coding for Gtfs has emerged as a novel treatment option for caries. Gong et al. developed a self-targeted CRISPR genome editing technology targeting virulence genes *gtfs*, which could effectively interfere with extracellular polysaccharide synthesis and biofilm formation [96]. Rajabi et al. reported that lytic bacteriophages could inhibit the expression of the *gtfs* gene, effectively suppressing the growth of *S. mutans* and biofilm formation [97]. Altogether, future studies should focus more on how bacteriophages and antimicrobial peptides regulate the virulence properties of cariogenic bacteria.

## Phage therapy for endodontic infections encounters both hurdles and opportunities

Endodontic infections are caused by pathogenic bacteria, among which *F. nucleatum* is more abundant in primary infections, while *Enterococcus faecalis* is more common in secondary infections. To be more precise, *E. faecalis* mediates the transformation from primary to secondary infection by physically binding *F. nucleatum* [98]. An exciting discovery was that endodontic bacterial communities with the same clinical disease had conserved metabolizing gene expression profiles with similar physiological and pathogenic behaviors [99]. Among the diverse endodontic diseases, refractory apical periodontitis (RAP) is characterized by persistent inflammation, progressive alveolar bone destruction, and delayed bone healing. *E. faecalis*, a significant pathogen of RAP, mediates inflammatory responses and modifies the polarization and differentiation of osteoblasts and macrophages [100].

### Continuous isolated Enterococcus bacteriophages: the promising countermeasure against antibiotic resistance

Antibiotics are traditionally applied in endodontic treatment, but there is growing concern about antibiotic resistance, particularly with the vancomycin-resistant *Enterococcus faecium* (VREfm) [101]. Notably, phage therapy is back in the picture again. In a recent review, Vehreschild et al. summarized the isolated anti-*E. faecalis* phages up to that point, showing their efficacy against *E. faecalis* within biofilms, especially the VRE strains [15]. Encouragingly, new bacteriophages against *E. faecalis* are still being isolated and characterized. For instance, vB_EfaS_LM99 [102] and vB_EfaS-SRH2 [103] have relatively narrow host ranges, while vB_EfaH_163 [104], vB_EfaS-Zip (Zip), and vB_EfaP-Max (Max) [105] exhibit a wide host lytic range, including both *E. faecalis* and *E. faecium*. In addition, vB_EfaS-271 [106,107] and vB_ZEFP [108] have been demonstrated to lyse *E. faecalis* in biofilms. Table 1 provides a detailed characterization of these bacteriophages.

**Table 1.** Bacteriophages and their characteristics in different oral diseases.

oral diseases	bacterium	phage name or proposed family	lysogenic/lytic	characterization	origin	references
Periodontal diseases	*Porphyromonas gingivalis*	*Alisviridae* (proposed)	lysogenic	insert into the host genome at non-specific sites by transposition	from the bacterial culture fluid	[66]
		*Ludisviridae* (proposed)	lysogenic	insert into the host’s tRNA-serine gene	from the bacterial culture fluid	[66]
		*Nixviridae* (proposed)	lysogenic	insert into the host’s tRNA-proline gene	from bacterial culture fluid	[66]
	*Tannerella forsythia*	*Heunggongvirae* (proposed)	lysogenic	–	from human supragingival plaque	[31]
	*Treponema denticola*	φtd1	lysogenic	circularised, double-stranded DNA, present attP site and Met-tRNA in the 3’ end	from the *T. denticola* biofilm cultured *in vitro*	[68]
	*Fusobacterium nucleatum*	FnpΦ02	lytic	has a narrow range, lyse slowly	from saliva samples (Santiago, Chile)	[70]
		FNU1	lytic	intracellularly lyses bacteria and significantly reduce biofilm mass	from mouthwash samples (Victoria, Australia)	[71]
	*Aggregatibacteractinomycetemcomitans*	AabΦ01	lytic	is specific for *A.a* serotype b, kills the bacteria within the biofilm	from clinical samples (Santiago, Chile)	[74]
		S1249	lysogenic	infects *A.a* serotype a and c, exists both lysogenic and pseudolysogenic cycles in bacteria	from clinical strain D11S–1	[80]
	*Actinomyces naeslundii*	Av-1	lytic	the lytic function gene consists of two holin-like genes holA and holB, and an endolysin gene lysA	from human gingival plaque (London)	[75,109]
	–	*Siphoviridae_*29632	unknown	has a higher detection rate in patients with chronic periodontitis than in healthy group	from periodontal pockets (Shanghai, China)	[76]
Caries	*Streptococcus mutans*	M102	lytic	specific for serotype c of *S. mutans*, owns an unusual lysis cassette containing two lytic enzymes	from saliva samples (Toulouse, France)	[88]
		ΦAPCM01	lytic	owns a narrow host range, reduces the metabolic activity of *S. mutans* biofilm	from human saliva samples (Basingstoke, United Kingdom)	[89]
		SMHBZ8	lytic	could penetrate, control, and inhibit the growth of *S. mutans* biofilm	from human saliva samples (MI, USA)	[90]
	*Actinomyces odontolyticus*	xhp1	lysogenic	the linear plasmid-like prophage does not integrate into the host genome or form any plaque in XH001	predicted from the XH001 genome	[92]
Endodontic infections	*Enterococcus faecalis*	IME-EF1	lytic	owns narrow host range, endolysin has a broader bactericidal spectrum, including two vancomycin-resistant strains of *E. faecalis*	from hospital sewage (Beijing, China)	[110]
		vB_EfaS_LM99	lytic	has broad host ranges, lyses one of *E. faecalis* isolates from implant-associated infections and facilitates external lysis of two others is tolerant to a wide range of environmental conditions	from a wastewater (Braga, Portugal)	[102]
		vB_EfaS-Zip (Zip)	lytic	has a wide host lytic range among clinical isolates with an average burst size of 38 PFU per infected cell	from sewage water (Frossos, Portuguesa)	[105]
		vB_EfaP-Max (Max)	lytic	has a wide host lytic range among clinical isolates with an average burst size of 52 PFU per infected cell	from sewage water (Frossos, Portuguesa)	[105]
		Idefix	lytic	most isolates of *E. faecalis* (including V583) are resistant to it	from sewage water sample (Paris, France)	[111]
		vB_EfaS-271	lytic	significantly reduces the number of *E. faecalis* in biofilm, emerges phage-resistant bacteria faster at lower M.O.I.	from urban sewage (Gdansk, Poland)	[106,107]
		vB_ZEFP	lytic	owns a wide host range with a burst size of 110 per infected cell, reduces the pre-formed bacterial biofilms	from sewage samples (Ismailia, Egypt)	[108]
		vB_EfaS-SRH2	lytic	only *E. faecalis* ATCC 29212 and clinical isolates of *E. faecalis* are susceptible to it	from wastewater samples (Isfahan, Iran)	[103]
		vB_EfaH_163	lytic	owns a wide host range involves *E. faecium* (includes 16 vancomycin-resistant clinical isolates) and some *E. faecalis strains* (includes a linezolid-resistant clinical isolate)	from human fecal samples (Gijón, Spain)	[104]
	*Enterococcus faecium*	IME-EFm5	lytic	has a narrow host range, only infects the *E. faecium* 4P-SA strain	from hospital sewage (Changchun, China)	[112]
		vB_EfaP_IME199	lytic	specifically infects *E. faecium*, has very low homology to all other known phage gene sequences.	from sewage water (Beijing, China)	[113]
		vB_EfaS-Zip (Zip)	lytic	has a wide host lytic range among clinical isolates with an average burst size of 38 PFU per infected cell	from sewage water (Frossos, Portuguesa)	[105]
		vB_EfaP-Max (Max)	lytic	has a wide host lytic range among clinical isolates with an average burst size of 52PFU per infected cell	from sewage water (Frossos, Portuguesa)	[105]
		vB_EfaH_163	lytic	the host range involves many *E. faecium* (includes 16 vancomycin-resistant clinical isolate), some *E. faecalis strains* (includes a linezolid-resistant clinical isolate)	from human fecal samples (Gijón, Spain)	[104]
		iF6	lytic	is specific to *E. faecium* and a single *E. thailandicus* strain, is stable only near pH 7	from the commercial phage cocktail ‘Sextaphag®’	[114]

Bothered by resistance to multiple antibiotics, research on *E. faecalis*-specific phages has yielded promising results, including vB_EfaP_IME199 [113] and the newly isolated iF6 [114]. The *E. faecalis* phages provide a vast array of therapeutic options because of their varieties in morphology, host range, and potential functions. For instance, vB_ZEFP could lyse *E. faecalis in vitro* and prevent *ex-vivo E. faecalis* root canal infection [108], indicating the great potential of bacteriophages in controlling root canal infections. In the phage vB_EfaH_163 gene, the coding region of a carbohydrate-binding protein is adjacent to a tail protein with a hydrolase structural domain, which facilitates viral DNA injection [104]. Moreover, the safety of bacteriophages has been demonstrated. Despite having highly effective lytic activity for bacteria colonizing the cells, Zip and Max showed no cytotoxicity to mammalian epithelial cells [105].

### New challenges in phage therapy for endodontic infections and how to deal with them

Unfortunately, bacteriophages in rapid evolution pose new challenges for clinical applications, i.e. phage resistance. EFDG1r, a mutant resistant to EFDG1, spawned the discovery of EFLK1. Furthermore, a combination of two bacteriophages (EFLK1 + EFDG1) could effectively lyse *E. faecalis* V583 strains regardless of antibiotic or bacteriophage resistance, with a 1:1 ratio phage cocktail showing the best results [115]. Thanks to the Abiα encoded by prophage 6, the *E. faecalis* V583 was resistant to the virulent bacteriophage Idefix, whereas mutations in the epa variable region served as an additional defense [111]. A similar mechanism of spontaneous phage resistance has been reported in *E. faecalis* OG1RF, in which the phage NPV1 adsorption incompetence was due to an *epaR* inactivation mutation [116].

Combinations of active bacteriophages in cocktail therapy are very valuable in preventing the rapid emergence of phage resistance. Using 47 collections of 20 *E. faecalis* phages, Wandro et al. found that mixtures of two or more different bacteriophages effectively reduced phage resistance mutants; a greater diversity of mixtures might increase the odds of phage antagonistic interactions [117]. The co-evolutionary arms race dynamic between bacteriophages and hosts generally gives rise to bacterial resistance, increasing phage infectivity over time. To maintain longer-lasting therapies, we should comprehensively characterize bacteriophages and select the combinations carefully.

The combination of antibiotics and bacteriophages may not only fight phage resistance but may also reverse antibiotic resistance. Shlezinger et al. reported that low vancomycin concentrations synergizing with bacteriophage EFLK1 could lyse VRE strains and disrupt biofilms [118]. This phage‒antibiotic synergy (PAS) delays the emergence of phage-resistant variants, possibly because bacteriophage infection enhances antibiotic sensitivity to specific binding sites [118]. Interestingly, Canfield et al. reported that phage 9183 mutations in *sagA*, *epaR*, and *epaX* could alter cell-surface phosphomimetic acids, enhancing *E. faecalis* sensitivity to ceftriaxone [119]. From this perspective, phage therapy appears to be a promising strategy to reverse antibiotic resistance in bacteria.

Lastly, endolysin provides a choice for phage therapy. In a mouse model, Cheng et al. found that the endolysin LysEF-P10 eliminated multidrug-resistant *E. faecalis* from the intestine without harming the intestinal microbiome [120]. Although IME-EFm5’s limited host range renders it unsuitable for clinical therapy, LysEFm5, its endolysin, exhibits a broader bactericidal spectrum encompassing seven strains of vancomycin-resistant *E. faecalis* [112]. However, it remains puzzling how endolysin could gain a broader host range than their parental bacteriophages. Other endolysins, two of iF6, Gp82, and Gp84, could lyse *E. faecalis* in both logarithmic and stable growth phases. In addition, the ubiquitinated Gp 84 endolysin could lyse 77% of the *E. faecalis* strains used in the experiment [114]. The efficacy of lysins, such as IME-EF1 endolysin [101] and Lys170 [102], has also been demonstrated in previous experiments. The spotlight of recent research is the structural and biochemical analysis of Lys170 [121,122], which might provide references for modifying or engineering secure, potent lysins that target *E. faecalis*.

## Phage vaccines: an option for oral cancer control

Many observational research studies and systematic reviews have suggested possible links between periodontal diseases and cancer. Chang et al. first demonstrated that bacteria in oral squamous cell carcinoma (OSCC) were related to the species and bacterial counts in subgingival plaque. In particular, *P. gingivalis* infection was associated with late clinical staging, low differentiation, lymph node metastasis, severe clinical attachment loss, etc [123]. Consequently, there is increasing awareness of the crucial role of periodontopathogenic bacteria in oral squamous cell carcinoma. Li et al. reported that *P. gingivalis*, *F. nucleatum*, and *T. denticola* could stimulate oral squamous cell carcinoma (OSCC) by promoting epithelial cell proliferation, inhibiting apoptosis, and modulating the inflammatory microenvironment [124]. Microbial dysbiosis may serve as a bridge between periodontal diseases and oral cancer [125], and the next question is whether viruses are involved in the progression. Castellsagué et al. provided convincing support that a significant proportion of oropharyngeal cancers (18.5‒22.4%) may be driven by HPV infection (primarily HPV 16) [126]. Apart from eukaryotic viruses, bacteriophages may also engage in the pathogenesis of oral cancer. As Podlach demonstrated, bacteriophages were prevalent in eukaryotic cells and could infiltrate human tissues through various pathways [127].

Notably, the functional peptides derived from bacteriophages have the advantages of high specificity for cancer cells and little immunogenicity in humans. Zhai et al. recently discovered that oral immunization with bacteriophage MS2-L2 VLPs could protect against oral infections with multiple HPV types associated with head and neck cancers, including HPV16, 35, 39, 52, and 58 [128]. This has significantly encouraged the creation of phage vaccines against oral cancers. Moreover, another possibility lies in the use and design of phage display peptides as delivery vehicles for chemotherapeutic drugs. They have the natural advantages of being usually non-immunogenic and easily penetrating tissues, which could specifically target cancer cells and improve the chemotherapeutic agents’ efficacy [129].

This table summarizes bacteriophages targeting different oral pathogens in terms of disease classification. Bacteriophages in periodontal diseases, caries, and endodontic diseases are described exhaustively, including their replication characteristics, isolation origins, host ranges, and specific gene function clusters.

## Conclusions and perspectives

The complex bacteria in the oral cavity play an integral role in the progression of various oral diseases. However, an increasing number of studies have demonstrated that bacteriophages might also be involved in the etiology of these infectious diseases. Here, we detail the evolutionary characterization of the complex dynamics among bacteriophages, bacteria, and eukaryotic cells in the oral ecological niche, particularly concerning the emergence of anti-bacteriophage resistance and the unique dynamics within oral biofilms. From the perspective of different oral diseases, we summarize the bacteriophages and their traits that target pathogenic bacteria with a role in periodontal diseases, caries, and endodontic infections.

However, with the widespread emergence of antibiotic resistance, the clinical use of phage therapy with high specificity and biosafety has become more urgent. The increasing success in separating lytic clinical bacteriophages *in vitro*, purifying lysins, and sequencing the bacterial pathogenicity islands in lysogenic bacteriophages are encouraging breakthroughs. In periodontal diseases, bacteriophages can be combined with conventional antibiotics to minimize adverse effects, while in caries, bacteriophages and their antimicrobial peptides or lysins, and even genetically editing virulence factors carried by prophages can be applied as therapeutic options. Current research on phage therapy for endodontic infections remains limited to *in vitro* and animal studies, thus little is known about its safety and efficacy in clinical practice. However, with the emergence of new problems such as phage resistance, how to choose the most secure and appropriate bacteriophages for cocktail therapy, the combination of antibiotics and bacteriophages, and endolysin have become new issues. Nonetheless, bacteriophages also suffer from shortcomings such as instability and short maintenance time. Future research can refer to phage encapsulation technology used in intestinal microbiome regulation to achieve the intelligent release of phages based on salivary pH changes. And additional hurdles for phage therapy include the inability of isolated phages to cope with all the complex and individualized clinical cases, the evolution of clinical bacterial strains, the variability and unpredictable of immune responses, the rapid emergence of phage resistance. Fortunately, with the advances in viral metagenomics and phage genome engineering, researchers would gain a deeper insight into the genetic properties of bacteriophages and further modify them according to clinical wishes.

Since the research focusing on bacteriophages and oral tumors has shown that bacteriophage MS2 is an excellent tool for producing targeted vaccines, we hypothesize that bacteriophages can also prevent oral diseases. In conclusion, in terms of etiology, bacteriophages have a strong relationship with the occurrence and regression of multiple oral diseases, and in terms of clinical applications, bacteriophages have potential value in the diagnosis, prevention, and treatment of oral diseases. To establish a solid foundation for the broad application of phage therapy in oral diseases, we strongly encourage further pertinent research to explore the infinite possibilities of bacteriophages in the oral cavity.

## References

[1] Kinane DF, Stathopoulou PG, Papapanou PN. Periodontal diseases. Nat Rev Dis Primers. 2017;3(1):17038. doi: 10.1038/nrdp.2017.38 28805207

[2] Lamont RJ, Koo H, Hajishengallis G. The oral microbiota: dynamic communities and host interactions. Nat Rev Microbiol. 2018;16(12):745–15. doi: 10.1038/s41579-018-0089-x 30301974 PMC6278837

[3] Radaic A, Kapila YL. The oralome and its dysbiosis: New insights into oral microbiome-host interactions. Comput Struct Biotechnol J. 2021;19:1335–1360. doi: 10.1016/j.csbj.2021.02.010 33777334 PMC7960681

[4] Baker JL, Mark Welch JL, Kauffman KM, et al. The oral microbiome: diversity, biogeography and human health. Nature Rev Microbiol. 2024;22(2):89–104. doi: 10.1038/s41579-023-00963-6 37700024 PMC11084736

[5] Abeles SR, Robles-Sikisaka R, Ly M, et al. Human oral viruses are personal, persistent and gender-consistent. Isme J. 2014;8(9):1753–1767. doi: 10.1038/ismej.2014.31 24646696 PMC4139723

[6] Baker JL, Bor B, Agnello M, et al. Ecology of the oral microbiome: beyond bacteria. Trends Microbiol. 2017;25(5):362–374. doi: 10.1016/j.tim.2016.12.012 28089325 PMC5687246

[7] Ly M, Abeles SR, Boehm TK, et al. Altered oral viral ecology in association with periodontal disease. MBio. 2014;5(3):e01133–14. doi: 10.1128/mBio.01133-14 24846382 PMC4030452

[8] Pride DT, Salzman J, Haynes M, et al. Evidence of a robust resident bacteriophage population revealed through analysis of the human salivary virome. Isme J. 2012;6(5):915–926. doi: 10.1038/ismej.2011.169 22158393 PMC3329113

[9] Liang G, Bushman FD. The human virome: assembly, composition and host interactions. Nature Rev Microbiol. 2021;19(8):514–527. doi: 10.1038/s41579-021-00536-5 33785903 PMC8008777

[10] Pinto G, Silva MD, Peddey M, et al. The role of bacteriophages in periodontal health and disease. Future Microbiol. 2016;11(10):1359–1369. doi: 10.2217/fmb-2016-0081 27633580

[11] Kowalski J, Górska R, Cieślik M, et al. What are the potential benefits of using bacteriophages in periodontal therapy? Antibiotics. 2022;11(4):446. doi: 10.3390/antibiotics11040446 35453197 PMC9027636

[12] Szafrański SP, Winkel A, Stiesch M. The use of bacteriophages to biocontrol oral biofilms. J Biotechnol. 2017;250:29–44. doi: 10.1016/j.jbiotec.2017.01.002 28108235

[13] Wolfoviz-Zilberman A, Kraitman R, Hazan R, et al. Phage targeting *Streptococcus mutans* in vitro and in vivo as a caries-preventive modality. Antibiotics. 2021;10(8):1015. doi: 10.3390/antibiotics10081015 34439064 PMC8389033

[14] Khalifa L, Brosh Y, Gelman D, et al. Targeting *Enterococcus faecalis* biofilms with phage therapy. Appl Environ Microbiol. 2015;81(8):2696–2705. doi: 10.1128/AEM.00096-15 25662974 PMC4375334

[15] Khalifa L, Shlezinger M, Beyth S, et al. Phage therapy against *Enterococcus faecalis* in dental root canals. J Oral Microbiol. 2016;8(1):32157. doi: 10.3402/jom.v8.32157 27640530 PMC5027333

[16] Shlezinger M, Friedman M, Houri-Haddad Y, et al. Phages in a thermoreversible sustained-release formulation targeting E. faecalis in vitro and in vivo. PloS One. 2019;14(7):e0219599. doi: 10.1371/journal.pone.0219599 31291645 PMC6620107

[17] Steier L, de Oliveira SD, de Figueiredo JAP. Bacteriophages in dentistry—state of the art and perspectives. Dent J (Basel). 2019;7(1):6. doi: 10.3390/dj7010006 30634460 PMC6473837

[18] Ly M, Jones MB, Abeles SR, et al. Transmission of viruses via our microbiomes. Microbiome. 2016;4(1). doi: 10.1186/s40168-016-0212-z PMC5134127 27912785

[19] Naidu M, Robles-Sikisaka R, Abeles SR, et al. Characterization of bacteriophage communities and CRISPR profiles from dental plaque. BMC Microbiol. 2014;14(1):175. doi: 10.1186/1471-2180-14-175 24981669 PMC4104742

[20] Pérez-Brocal V, Moya A. The analysis of the oral DNA virome reveals which viruses are widespread and rare among healthy young adults in Valencia (Spain). PLOS ONE. 2018;13(2):e0191867. doi: 10.1371/journal.pone.0191867 29420668 PMC5805259

[21] Weinbauer MG. Ecology of prokaryotic viruses. FEMS Microbiol Rev. 2004;28(2):127–181. doi: 10.1016/j.femsre.2003.08.001 15109783

[22] Oliveira H, São-José C, Azeredo J. Phage-derived peptidoglycan degrading enzymes: challenges and future prospects for in vivo therapy. Viruses. 2018;10(6):292. doi: 10.3390/v10060292 29844287 PMC6024856

[23] Mirzaei MK, Maurice CF. Menage a trois in the human gut: interactions between host, bacteria and phages. Nat Rev Microbiol. 2017;15(7):397–408. doi: 10.1038/nrmicro.2017.30 28461690

[24] Canchaya C, Fournous G, Chibani-Chennoufi S, et al. Phage as agents of lateral gene transfer. Curr Opin Microbiol. 2003;6(4):417–424. doi: 10.1016/S1369-5274(03)00086-9 12941415

[25] Abeles SR, Pride DT. Molecular bases and role of viruses in the human microbiome. J Mol Biol. 2014;426(23):3892–3906. doi: 10.1016/j.jmb.2014.07.002 25020228 PMC7172398

[26] Teklemariam AD, Al-Hindi RR, Qadri I, et al. The battle between bacteria and bacteriophages: a conundrum to their immune system. Antibiotics. 2023;12(2):381. doi: 10.3390/antibiotics12020381 36830292 PMC9952470

[27] Hasan M, Ahn J. Evolutionary dynamics between phages and bacteria as a possible approach for designing effective phage therapies against antibiotic-resistant bacteria. Antibiotics. 2022;11(7):915. doi: 10.3390/antibiotics11070915 35884169 PMC9311878

[28] Safari F, Sharifi M, Farajnia S, et al. The interaction of phages and bacteria: the co-evolutionary arms race. Crit Rev Biotechnol. 2020;40(2):119–137. doi: 10.1080/07388551.2019.1674774 31793351

[29] Hampton HG, Watson BNJ, Fineran PC. The arms race between bacteria and their phage foes. Nature. 2020;577(7790):327–336. doi: 10.1038/s41586-019-1894-8 31942051

[30] Malone LM, Birkholz N, Fineran PC. Conquering CRISPR: how phages overcome bacterial adaptive immunity. Curr Opin Biotechnol. 2021;68:30–36. doi: 10.1016/j.copbio.2020.09.008 33113496

[31] Szafranski SP, Slots J, Stiesch M. The human oral phageome. Periodontol 2000. 2021;86(1):79–96. doi: 10.1111/prd.12363 33690937

[32] Zou G, He L, Rao J, et al. Improving the safety and efficacy of phage therapy from the perspective of phage-mammal interactions. FEMS Microbiol Rev. 2023;47(4). doi: 10.1093/femsre/fuad042 37442611

[33] Hansen MF, Svenningsen SL, Røder HL, et al. Big impact of the tiny: bacteriophage–bacteria interactions in biofilms. Trends Microbiol. 2019;27(9):739–752. doi: 10.1016/j.tim.2019.04.006 31128928

[34] Correa AMS, Howard-Varona C, Coy SR, et al. Revisiting the rules of life for viruses of microorganisms. Nature Rev Microbiol. 2021;19(8):501–513. doi: 10.1038/s41579-021-00530-x 33762712

[35] Peng X, Cheng L, You Y, et al. Oral microbiota in human systematic diseases. Int J Oral Sci. 2022;14(1):14. doi: 10.1038/s41368-022-00163-7 35236828 PMC8891310

[36] Bull JJ, Christensen K, Scott C, et al. Phage-bacterial dynamics with spatial structure: self organization around phage sinks can promote increased cell densities. Antibiotics. 2018;7(1):8. doi: 10.3390/antibiotics7010008 29382134 PMC5872119

[37] Silpe JE, Bassler BL. A host-produced quorum-sensing autoinducer controls a phage lysis-lysogeny decision. Cell. 2019;176(1–2):268–280.e13. doi: 10.1016/j.cell.2018.10.059 30554875 PMC6329655

[38] Abedon ST. Ecology and evolutionary biology of hindering phage therapy: the phage tolerance vs. phage resistance of bacterial biofilms. Antibiotics. 2023;12(2):245. doi: 10.3390/antibiotics12020245 36830158 PMC9952518

[39] Willner D, Furlan M, Schmieder R, et al. Metagenomic detection of phage-encoded platelet-binding factors in the human oral cavity. Proc Natl Acad Sci, USA. 2011;108(supplement_1):4547–4553. doi: 10.1073/pnas.1000089107 20547834 PMC3063595

[40] Shlezinger M, Khalifa L, Houri-Haddad Y, et al. Phage therapy: a new horizon in the antibacterial treatment of oral pathogens. Curr Top Med Chem. 2017;17(10):1199–1211. doi: 10.2174/1568026616666160930145649 27770768

[41] Martínez A, Kuraji R, Kapila YL. The human oral virome: Shedding light on the dark matter. Periodontol 2000. 2021;87(1):282–298. doi: 10.1111/prd.12396 34463988 PMC8457075

[42] Azam AH, Tan X-E, Veeranarayanan S, et al. Bacteriophage technology and modern medicine. Antibiotics. 2021;10(8):999. doi: 10.3390/antibiotics10080999 34439049 PMC8388951

[43] Gangwar M, Rastogi S, Singh D, et al. Immunological and safety profile of bacteriophage therapy: A pre-clinical study. J Appl Microbiol. 2022;133(3):1446–1460. doi: 10.1111/jam.15642 35633293

[44] Rappo U, Kahan-Hanum M, Ussery X, et al. 126 a phase 1b/2a randomized, double-blind, placebo-controlled, multicenter study evaluating nebulized phage therapy in people with cystic fibrosis with chronic Pseudomonas aeruginosa pulmonary infection. J Cystic Fibrosis. 2023;22:S66–S67. doi: 10.1016/S1569-1993(23)01059-7

[45] Chan B, Kortright K, Stanley G, et al. 41 CYstic Fibrosis bacterioPhage study at Yale (CYPHY). J Cystic Fibrosis. 2023;22:22. doi: 10.1016/S1569-1993(23)00976-1

[46] Sarker SA, Sultana S, Reuteler G, et al. Oral phage therapy of acute bacterial diarrhea with two coliphage preparations: a randomized trial in children from Bangladesh. EBioMedicine. 2016;4:124–137. doi: 10.1016/j.ebiom.2015.12.023 26981577 PMC4776075

[47] Sarker SA, Berger B, Deng Y, et al. Oral application of Escherichia coli bacteriophage: safety tests in healthy and diarrheal children from Bangladesh. Environ Microbiol. 2017;19(1):237–250. doi: 10.1111/1462-2920.13574 27750388

[48] Karn SL, Bhartiya SK, Pratap A, et al. A randomized, placebo-controlled, double-blind clinical trial of bacteriophage cocktails in chronic wound infections. Int J Low Extrem Wounds. 2024:15347346231226342. doi: 10.1177/15347346231226342 38233034

[49] Jault P, Leclerc T, Jennes S, et al. Efficacy and tolerability of a cocktail of bacteriophages to treat burn wounds infected by Pseudomonas aeruginosa (PhagoBurn): a randomised, controlled, double-blind phase 1/2 trial. Lancet Infect Dis. 2019;19(1):35–45. doi: 10.1016/S1473-3099(18)30482-1 30292481

[50] Fedorov E, Samokhin A, Kozlova Y, et al. Short-term outcomes of phage-antibiotic combination treatment in adult patients with periprosthetic hip joint infection. Viruses. 2023;15(2):499. doi: 10.3390/v15020499 36851713 PMC9964274

[51] Doub JB, Ng VY, Wilson E, et al. Successful treatment of a Recalcitrant Staphylococcus epidermidis prosthetic knee infection with intraoperative bacteriophage therapy. Pharmaceuticals. 2021;14(3):231. doi: 10.3390/ph14030231 33800146 PMC7998749

[52] Wortelboer K, de Jonge PA, Scheithauer TPM, et al. Phage-microbe dynamics after sterile faecal filtrate transplantation in individuals with metabolic syndrome: a double-blind, randomised, placebo-controlled clinical trial assessing efficacy and safety. Nat Commun. 2023;14(1). doi: 10.1038/s41467-023-41329-z PMC10497675 37699894

[53] Michelson D, Grundman M, Magnuson K, et al. Randomized, placebo controlled trial of Npt088, a phage-derived, amyloid-targeted treatment for Alzheimer’s disease. J prev Alzheimer’s dis. 2019:1–4. doi: 10.14283/jpad.2019.37 31686093

[54] Hesse S, Adhya S. Phage therapy in the twenty-first century: facing the decline of the antibiotic era; is it finally time for the age of the phage? Annu Rev Microbiol. 2019;73(1):155–174. doi: 10.1146/annurev-micro-090817-062535 31185183

[55] Hatfull GF, Dedrick RM, Schooley RT. Phage therapy for antibiotic-resistant bacterial infections. Annu Rev Med. 2022;73(1):197–211. doi: 10.1146/annurev-med-080219-122208 34428079

[56] Barclay CW, Spence D, Laird WRE. Intra‐oral temperatures during function. J Oral Rehabil. 2005;32(12):886–894. doi: 10.1111/j.1365-2842.2005.01509.x 16297035

[57] Banar M, Rokaya D, Azizian R, et al. Oral bacteriophages: metagenomic clues to interpret microbiomes. PeerJ. 2024;12:12. doi: 10.7717/peerj.16947 PMC10885796 38406289

[58] Michaud DS, Fu Z, Shi J, et al. Periodontal disease, tooth loss, and cancer risk. Epidemiol Rev. 2017;39(1):49–58. doi: 10.1093/epirev/mxx006 28449041 PMC5868279

[59] Maisonneuve P, Amar S, Lowenfels AB. Periodontal disease, edentulism, and pancreatic cancer: a meta-analysis. Ann Oncol. 2017;28(5):985–995. doi: 10.1093/annonc/mdx019 28453689

[60] Loos BG, Van Dyke TE. The role of inflammation and genetics in periodontal disease. Periodontol 2000. 2020;83(1):26–39. doi: 10.1111/prd.12297 32385877 PMC7319430

[61] Chen C, Feng P, Slots J. Herpesvirus-bacteria synergistic interaction in periodontitis. Periodontol 2000. 2020;82(1):42–64. doi: 10.1111/prd.12311 31850623 PMC7382446

[62] Imai K, Ogata Y. How does Epstein–Barr Virus contribute to chronic periodontitis? IJMS. 2020;21(6):1940. doi: 10.3390/ijms21061940 32178406 PMC7139403

[63] Contreras A, Botero JE, Slots J. Biology and pathogenesis of cytomegalovirus in periodontal disease. Periodontol 2000. 2014;64(1):40–56. doi: 10.1111/j.1600-0757.2012.00448.x 24320955 PMC7167941

[64] Yost S, Duran-Pinedo AE, Teles R, et al. Functional signatures of oral dysbiosis during periodontitis progression revealed by microbial metatranscriptome analysis. Genome Med. 2015;7(1). doi: 10.1186/s13073-015-0153-3 PMC4410737 25918553

[65] Hajishengallis G, Darveau RP, Curtis MA. The keystone-pathogen hypothesis. Nat Rev Microbiol. 2012;10(10):717–725. doi: 10.1038/nrmicro2873 22941505 PMC3498498

[66] Matrishin CB, Haase EM, Dewhirst FE, et al. Phages are unrecognized players in the ecology of the oral pathogen Porphyromonas gingivalis. Microbiome. 2023;11(1). doi: 10.1186/s40168-023-01607-w PMC10367356 37491415

[67] Holt SC, Ebersole JL. Porphyromonas gingivalis, Treponema denticola, and Tannerella forsythia: the ‘red complex’, a prototype polybacterial pathogenic consortium in periodontitis. Periodontol 2000. 2005;38(1):72–122. doi: 10.1111/j.1600-0757.2005.00113.x 15853938

[68] Mitchell HL, Dashper SG, Catmull DV, et al. Treponema denticola biofilm-induced expression of a bacteriophage, toxin–antitoxin systems and transposases. Microbiology. 2010;156(3):774–788. doi: 10.1099/mic.0.033654-0 20007650

[69] Brennan CA, Garrett WS. *Fusobacterium nucleatum* - symbiont, opportunist and oncobacterium. Nat Rev Microbiol. 2019;17(3):156–166. doi: 10.1038/s41579-018-0129-6 30546113 PMC6589823

[70] Machuca P, Daille L, Vinés E, et al. Isolation of a novel bacteriophage specific for the periodontal pathogen Fusobacterium nucleatum. Appl Environ Microbiol. 2010;76(21):7243–7250. doi: 10.1128/AEM.01135-10 20851973 PMC2976222

[71] Kabwe M, Brown TL, Dashper S, et al. Genomic, morphological and functional characterisation of novel bacteriophage FNU1 capable of disrupting *Fusobacterium nucleatum* biofilms. Sci Rep. 2019;9(1):9107. doi: 10.1038/s41598-019-45549-6 31235721 PMC6591296

[72] Darveau RP. Periodontitis: a polymicrobial disruption of host homeostasis. Nat Rev Microbiol. 2010;8(7):481–490. doi: 10.1038/nrmicro2337 20514045

[73] Vega BA, Belinka BA Jr., Kachlany SC. Aggregatibacter actinomycetemcomitans Leukotoxin (LtxA; Leukothera®): Mechanisms of action and therapeutic applications. Toxins (Basel). 2019;11(9):489. doi: 10.3390/toxins11090489 31454891 PMC6784247

[74] Castillo-Ruiz M, Vinés ED, Montt C, et al. Isolation of a Novel Aggregatibacter actinomycetemcomitans Serotype b bacteriophage capable of Lysing Bacteria within a biofilm. Appl environ microbiol. 2011;77(9):3157–3159. doi: 10.1128/AEM.02115-10 21378052 PMC3126385

[75] Delisle AL, Barcak GJ, Guo M. Isolation and expression of the lysis genes of actinomyces naeslundii phage av-1. Appl Environ Microbiol. 2006;72(2):1110–1117. doi: 10.1128/AEM.72.2.1110-1117.2006 16461656 PMC1392916

[76] Zhang Y, Shan T-L, Li F, et al. A novel phage from periodontal pockets associated with chronic periodontitis. Vir Gen. 2019;55(3):381–393. doi: 10.1007/s11262-019-01658-y 30927185

[77] Endo A, Watanabe T, Ogata N, et al. Comparative genome analysis and identification of competitive and cooperative interactions in a polymicrobial disease. Isme J. 2015;9(3):629–642. doi: 10.1038/ismej.2014.155 25171331 PMC4331577

[78] Yokogawa T, Nagano K, Fujita M, et al. Characterization of a Treponema denticola ATCC 35405 mutant strain with mutation accumulation, including a lack of phage-derived genes. PloS One. 2022;17(6):e0270198. doi: 10.1371/journal.pone.0270198 35749516 PMC9231711

[79] Szafranski SP, Kilian M, Yang I, et al. Diversity patterns of bacteriophages infecting aggregatibacter and Haemophilus species across clades and niches. Isme J. 2019;13(10):2500–2522. doi: 10.1038/s41396-019-0450-8 31201356 PMC6776037

[80] Tang-Siegel GG, Chen C, Mintz KP. Increased sensitivity of Aggregatibacter actinomycetemcomitans to human serum is mediated by induction of a bacteriophage. Mol Oral Microbiol. 2023;38(1):58–70. doi: 10.1111/omi.12378 35833243 PMC10087258

[81] Wang J, Gao Y, Zhao F. Phage-bacteria interaction network in human oral microbiome. Environ Microbiol. 2016;18(7):2143–2158. doi: 10.1111/1462-2920.12923 26036920

[82] Ferri M, Ranucci E, Romagnoli P, et al. Antimicrobial resistance: a global emerging threat to public health systems. Crit Rev Food Sci Nutr. 2017;57(13):2857–2876. doi: 10.1080/10408398.2015.1077192 26464037

[83] Abdulkareem A, Abdulbaqi H, Gul S, et al. Classic vs. Novel antibacterial approaches for eradicating dental biofilm as adjunct to periodontal debridement: an evidence-based overview. Antibiotics. 2021;11(1):9. doi: 10.3390/antibiotics11010009 35052887 PMC8773342

[84] Amankwah S, Abdella K, Kassa T. Bacterial biofilm destruction: a focused review on the recent use of phage-based strategies with other antibiofilm agents. Nanotechnol Sci Appl. 2021;14:161–177. doi: 10.2147/NSA.S325594 34548785 PMC8449863

[85] Yang Y, Du H, Zou G, et al. Encapsulation and delivery of phage as a novel method for gut flora manipulation in situ: A review. J Controlled Release. 2023;353:634–649. doi: 10.1016/j.jconrel.2022.11.048 36464065

[86] Jakubovics NS, Yassin SA, Rickard AH. Community Interactions of Oral Streptococci. Adv Appl Microbiol. 2014(87):43–110. 24581389 10.1016/B978-0-12-800261-2.00002-5

[87] Baker JL, Morton JT, Dinis M, et al. Deep metagenomics examines the oral microbiome during dental caries, revealing novel taxa and co-occurrences with host molecules. Genome Res. 2021;31(1):64–74. doi: 10.1101/gr.265645.120 33239396 PMC7849383

[88] van der Ploeg JR. Genome sequence of *Streptococcus mutans* bacteriophage M102. FEMS Microbiol Lett. 2007;275(1):130–138. doi: 10.1111/j.1574-6968.2007.00873.x 17711456

[89] Dalmasso M, de Haas E, Neve H, et al. Isolation of a novel phage with activity against *Streptococcus mutans* biofilms. PloS One. 2015;10(9):e0138651. doi: 10.1371/journal.pone.0138651 26398909 PMC4580409

[90] Ben-Zaken H, Kraitman R, Coppenhagen-Glazer S, et al. Isolation and characterization of *Streptococcus mutans* phage as a possible treatment agent for caries. Viruses. 2021;13(5):825. doi: 10.3390/v13050825 34063251 PMC8147482

[91] Tang G, Samaranayake LP, Yip H-K, et al. Direct detection of Actinomyces spp. From infected root canals in a Chinese population: a study using PCR-based, oligonucleotide-DNA hybridization technique. J Dent. 2003;31(8):559–568. doi: 10.1016/S0300-5712(03)00112-X 14554073

[92] Shen M, Yang Y, Shen W, et al. A linear plasmid-like prophage of actinomyces odontolyticus promotes biofilm assembly. Appl Environ Microbiol. 2018;84(17). doi: 10.1128/AEM.01263-18 PMC6102993 29915115

[93] Guo L, McLean JS, Yang Y, et al. Precision-guided antimicrobial peptide as a targeted modulator of human microbial ecology. Proc Natl Acad Sci U S A. 2015;112(24):7569–7574. doi: 10.1073/pnas.1506207112 26034276 PMC4475959

[94] Xu J, Yang H, Bi Y, et al. Activity of the chimeric lysin ClyR against common gram-positive oral microbes and its anticaries efficacy in rat models. Viruses. 2018;10(7):380. doi: 10.3390/v10070380 30036941 PMC6070986

[95] Jiang W, Wang Y, Luo J, et al. Antimicrobial Peptide GH12 Prevents Dental Caries by Regulating Dental Plaque Microbiota. Appl Environ Microbiol. 2020;86(14). doi: 10.1128/AEM.00527-20 PMC7357485 32414800

[96] Gong T, Tang B, Zhou X, et al. Genome editing in *Streptococcus mutans* through self-targeting CRISPR arrays. Mol Oral Microbiol. 2018;33(6):440–449. doi: 10.1111/omi.12247 30329221

[97] Rajabi Z, Soltan Dallal MM, Afradi MR, et al. Comparison of the effect of extracted bacteriocin and lytic bacteriophage on the expression of biofilm associated genes in Streptococcus mutans. Adv Mater Sci Eng. 2022;2022:1–7. doi: 10.1155/2022/5035280

[98] Xiang D, Dong P-T, Cen L, et al. Antagonistic interaction between two key endodontic pathogens *Enterococcus faecalis* and Fusobacterium nucleatum. J Oral Microbiol. 2023;15(1):2149448. doi: 10.1080/20002297.2022.2149448 36452179 PMC9704101

[99] Siqueira JF Jr., Rocas IN. Present status and future directions: Microbiology of endodontic infections. Int Endod J. 2022;55(S3):512–530. doi: 10.1111/iej.13677 34958494

[100] Deng Z, Lin B, Liu F, et al. Role of *Enterococcus faecalis* in refractory apical periodontitis: from pathogenicity to host cell response. J Oral Microbiol. 2023;15(1):2184924. doi: 10.1080/20002297.2023.2184924 36891193 PMC9987735

[101] Vehreschild MJGT, Haverkamp M, Biehl LM, et al. Vancomycin-resistant enterococci (VRE): a reason to isolate? Infection. 2019;47(1):7–11. doi: 10.1007/s15010-018-1202-9 30178076

[102] Barros J, Melo LDR, Poeta P, et al. Lytic bacteriophages against multidrug-resistant Staphylococcus aureus, *Enterococcus faecalis* and Escherichia coli isolates from orthopaedic implant-associated infections. Int J Antimicrob Agents. 2019;54(3):329–337. doi: 10.1016/j.ijantimicag.2019.06.007 31229670

[103] Pazhouhnia S, Bouzari M, Arbabzadeh-Zavareh F. Isolation, characterization and complete genome analysis of a novel bacteriophage vB_efaS-SRH2 against *Enterococcus faecalis* isolated from periodontitis patients. Sci Rep. 2022;12(1). doi: 10.1038/s41598-022-16939-0 PMC9346004 35918375

[104] Pradal I, Casado A, Del Rio B, et al. Enterococcus faecium Bacteriophage vB_efah_163, a new member of the Herelleviridae family, reduces the mortality associated with an E. faecium vanR clinical isolate in a Galleria mellonella animal model. Viruses. 2023;15(1):179. doi: 10.3390/v15010179 36680219 PMC9860891

[105] Melo LDR, Ferreira R, Costa AR, et al. Efficacy and safety assessment of two enterococci phages in an in vitro biofilm wound model. Sci Rep. 2019;9(1). doi: 10.1038/s41598-019-43115-8 PMC6491613 31040333

[106] Topka-Bielecka G, Bloch S, Nejman-Faleńczyk B, et al. Characterization of the Bacteriophage vB_efaS-271 Infecting Enterococcus faecalis. IJMS. 2020;21(17):6345. doi: 10.3390/ijms21176345 32882938 PMC7503890

[107] Topka-Bielecka G, Nejman-Faleńczyk B, Bloch S, et al. Phage–bacteria interactions in potential applications of bacteriophage vB_efaS-271 against *Enterococcus faecalis*. Viruses. 2021;13(2):318. doi: 10.3390/v13020318 33669643 PMC7922982

[108] El-Telbany M, El-Didamony G, Askora A, et al. Bacteriophages to control multi-drug resistant *Enterococcus faecalis* infection of dental root canals. Microorganisms. 2021;9(3):517. doi: 10.3390/microorganisms9030517 33802385 PMC7998577

[109] Delisle AL, Nauman RK, Minah GE. Isolation of a bacteriophage for actinomyces viscosus. Infect Immun. 1978;20(1):303–306. doi: 10.1128/iai.20.1.303-306.1978 669798 PMC421850

[110] Zhang W, Mi Z, Yin X, et al. Characterization of *Enterococcus faecalis* phage IME-EF1 and its endolysin. PloS One. 2013;8(11):e80435. doi: 10.1371/journal.pone.0080435 24236180 PMC3827423

[111] Lossouarn J, Briet A, Moncaut E, et al. *Enterococcus faecalis* Countermeasures Defeat a Virulent Picovirinae Bacteriophage. Viruses. 2019;11(1):48. doi: 10.3390/v11010048 30634666 PMC6356687

[112] Gong P, Cheng M, Li X, et al. Characterization of Enterococcus faecium bacteriophage IME-EFm5 and its endolysin LysEFm5. Virology. 2016;492:11–20. doi: 10.1016/j.virol.2016.02.006 26896930

[113] Xing S, Zhang X, Sun Q, et al. Complete genome sequence of a novel, virulent Ahjdlikevirus bacteriophage that infects Enterococcus faecium. Arch Virol. 2017;162(12):3843–3847. doi: 10.1007/s00705-017-3503-1 28812171

[114] Buzikov RM, Kazantseva OA, Piligrimova EG, et al. Bacteriolytic potential of Enterococcus Phage iF6 Isolated from “Sextaphag(®)” Therapeutic Phage Cocktail and properties of its endolysins, Gp82 and Gp84. Viruses. 2023;15(3):767. 36992476 10.3390/v15030767 PMC10054541

[115] Khalifa L, Gelman D, Shlezinger M, et al. Defeating Antibiotic- and Phage-Resistant *Enterococcus faecalis* Using a Phage Cocktail in vitro and in a Clot Model. Front Microbiol. 2018;9:9. doi: 10.3389/fmicb.2018.00326 29541067 PMC5835721

[116] Ho K, Huo W, Pas S, et al. Loss-of-Function Mutations in epaR Confer Resistance to ϕNPV1 Infection in *Enterococcus faecalis* OG1RF. Antimicrob Agents Chemother. 2018;62(10). doi: 10.1128/AAC.00758-18 PMC6153818 30104266

[117] Wandro S, Ghatbale P, Attai H, et al. Phage Cocktails can Prevent the Evolution of Phage-Resistant Enterococcus.

[118] Shlezinger M, Coppenhagen-Glazer S, Gelman D, et al. Eradication of Vancomycin-Resistant Enterococci by Combining Phage and Vancomycin. Viruses. 2019;11(10):954. doi: 10.3390/v11100954 31623253 PMC6833023

[119] Canfield GS, Chatterjee A, Espinosa J, et al. Lytic Bacteriophages Facilitate Antibiotic Sensitization of Enterococcus faecium. Antimicrob Agents Chemother. 2021;65(5). doi: 10.1128/AAC.00143-21 PMC8092871 33649110

[120] Cheng M, Zhang Y, Li X, et al. Endolysin LysEF-P10 shows potential as an alternative treatment strategy for multidrug-resistant *Enterococcus faecalis* infections. Sci Rep. 2017;7(1):10164. doi: 10.1038/s41598-017-10755-7 28860505 PMC5579260

[121] Proença D, Velours C, Leandro C, et al. A two-component, multimeric endolysin encoded by a single gene. Mol Microbiol. 2015;95(5):739–753. doi: 10.1111/mmi.12857 25388025

[122] Xu X, Zhang D, Zhou B, et al. Structural and biochemical analyses of the tetrameric cell binding domain of Lys170 from enterococcal phage F170/08. Eur Biophys J. 2021;50(5):721–729. doi: 10.1007/s00249-021-01511-x 33609147

[123] Chang C, Geng F, Shi X, et al. The prevalence rate of periodontal pathogens and its association with oral squamous cell carcinoma. Appl Microbiol Biotechnol. 2019;103(3):1393–1404. doi: 10.1007/s00253-018-9475-6 30470868

[124] Li R, Xiao L, Gong T, et al. Role of oral microbiome in oral oncogenesis, tumor progression, and metastasis. Mol Oral Microbiol. 2023;38(1):9–22. doi: 10.1111/omi.12403 36420924

[125] Nwizu N, Wactawski‐Wende J, Genco RJ. Periodontal disease and cancer: Epidemiologic studies and possible mechanisms. Periodontol 2000. 2020;83(1):213–233. doi: 10.1111/prd.12329 32385885 PMC7328760

[126] Castellsagué X, Alemany L, Quer M, et al. HPV Involvement in Head and Neck Cancers: Comprehensive Assessment of Biomarkers in 3680 Patients. JNCI. 2016;108(6):djv403. doi: 10.1093/jnci/djv403 26823521

[127] Podlacha M, Grabowski Ł, Kosznik-Kawśnicka K, et al. Interactions of bacteriophages with animal and human organisms—safety issues in the light of phage therapy. Int J Mol Sci. 2021;22(16):8937. doi: 10.3390/ijms22168937 34445641 PMC8396182

[128] Zhai L, Yadav R, Kunda NK, et al. Oral immunization with bacteriophage MS2-L2 VLPs protects against oral and genital infection with multiple HPV types associated with head & neck cancers and cervical cancer. Antiviral Res. 2019;166:56–65. doi: 10.1016/j.antiviral.2019.03.012 30926288 PMC6538018

[129] Ghosh D, Peng X, Leal J, et al. Peptides as drug delivery vehicles across biological barriers. J Pharm Invest. 2018;48(1):89–111. doi: 10.1007/s40005-017-0374-0 PMC6023411 29963321

